# Some Lesser-Known Activities of the General Medical Council
*Based on the Presidential Address to the Bristol Medico-Chirurgical Society, 12 October 1960.


**Published:** 1961-07

**Authors:** R. J. Brocklehurst


### SOME LESSER-KNOWN ACTIVITIES OF THE GENERAL MEDICAL COUNCIL*

BY

PROFESSOR R. J. BROCKLEHURST, M.A., D.M., B.CH.

Two years ago the General Medical Council celebrated its Centenary. Its disciplinary and, to a smaller extent, its educational functions are well known; but I decided it might be appropriate if I dealt with some of its other, and lesser known, activities—at least in recent years.

### ORIGIN OF THE COUNCIL

The Medical Act of 1858 was designed primarily for the benefit of the public; it began: "Whereas it is expedient that persons requiring medical aid should be enabled to distinguish qualified from unqualified practitioners, be it therefore enacted . . ." The General Medical Council, or, as it was officially called until 10 years ago, "The General Council of Medical Education and Registration of the United Kingdom", was set up under this Act to safeguard the public interest and not to advance the welfare of the profession.

It was early pointed out that, for the Act to achieve its object, the legally qualified practitioner must be a well qualified practitioner, and the Council from its beginning was well aware that its chief duty was to establish and ensure proper educational standards for the profession. Its second main duty was to compile and keep up-to-date the Register of qualified practitioners, admitting to it only names of those whose education was deemed sufficient, and removing from it the names of those who had died and those guilty of criminal offences or of serious professional misconduct.

Constitutionally the G.M.C. was the first statutory tribunal of its kind with quasi-judicial powers over a whole profession; it is answerable to the Privy Council. It took several years for it to "find its feet" and discover the nature of its corporate powers; and the minutes of its earlier years surprise the present-day reader with the length and intricacy of the discussions which took place on a vast number of matters of fine detail. One can only judge that its members were a good deal more verbose in those days than they are now, and that they had more time to spare; but the present Council has the advantage of 100 years of experience and practice behind it and has no excuse for taking a long time to reach decisions.

It has been said by an historian that the development of a corporate spirit within the Council was not notable during the first half of its existence; many of its members were men of forceful character and strong opinions; and reading between the lines one can conjure up pictures of heated debates, not devoid of personal animosity. A favourite right, often insisted upon by the warmer-blooded members on finding themselves in a minority on a straight vote, was to demand the recording in the minutes of the names of those who had voted for and those who had voted against a motion. From the frequency of this procedure, and the names of those who demanded it, one can quite easily guess who were the chief firebrands. Nowadays, debates in the Council are peaceful, and the names of those voting for or against a motion are not recorded.

Sir Donald Macalister, who was President from 1904 to 1931, was a strong chairman, and inculcated discipline into the members. I was told by some who had sat under him that he developed the practice of personally advising new members not to speak in the Council Chamber for their first two years! Sir Norman Walker, his successor,

*Based on the Presidential Address to the Bristol Medico-Chirurgical Society, 12 October 1960.

on one occasion stated that "Sir Donald's influence somehow tended to discourage long speeches". It is noticeable however that nowadays new members quite often contribute to debates at their first session; for in fact succeeding Presidents have not exercised the same autocratic influence.

### COMPOSITION OF THE COUNCIL

The greater part of the G.M.C.'s membership, which now totals forty-seven, has always consisted of representatives of the Medical Licensing Bodies, i.e. the Universities which grant medical degrees and the Medical Corporations which grant diplomas. But when the Council was first set up the Act laid down that six of its members should be appointed by the Crown—four from England and one each from Scotland and Ireland. The Act of 1886 brought about a change; the Crown appointments were reduced to five, and provision was made for five direct representatives of the profession itself—three selected by practitioners in England and one each by practitioners in Scotland and Ireland. In 1950 the "non-academic" proportion was further increased. The Crown now appoints five registered medical practitioners (three from England and Wales, one from Scotland and one from Northern Ireland) and three lay people (two from England and Wales and one from Scotland); while the profession itself now elects eleven representatives—eight by practitioners in England and Wales, of whom one must be a practitioner in Wales, two by Scottish practitioners and one by practitioners in Ireland.

The lay appointments are always interesting to the medical members of the Council. It is recorded that Bernard Shaw claimed some credit for the intention that a member of the public should maintain a critical vigilance over the Council, especially in matters of discipline, and should represent the "consumer interest". Lord Nathan, a solicitor, who retired last year after ten years' membership, said regarding his experience on the Disciplinary Committee: "I found that work most interesting, the more so as I often felt that my medical colleagues were perhaps somewhat gentler than I myself might have been; they thus falsified, to their credit, an opinion which, mistakenly but I believe pretty generally, had prevailed that a lay member on the Disciplinary Committee was a safeguard to practitioners lest their fellows should be unduly harsh".

During my experience the lay members until recently have been parliamentarians: Lord Nathan, from the House of Lords; Sir Douglas Hacking, Mr. J. J. Robertson, Miss Florence Horsbrugh, Mr. J. B. Hynd and Sir William Anstruther Gray, from the Commons. But two of the present lay members are not members of either house: Dame Myra Curtis, previously head of one of the women's colleges at Cambridge; and Mr. C. W. Guillebaud, a member of the same University, whose name is familiar to those concerned with the Health Services of this country.

In 1950 a woman, in the person of Dame Hilda Lloyd, was appointed to represent the Royal College of Obstetricians and Gynaecologists. Although Dame Hilda was not actually the first woman member, for Dr. Christine Murrell was elected as a direct representative in 1933, she was the first to participate in the duties of membership as Dr. Murrell unfortunately died before she could take her seat. If Dame Myra Curtis felt lonely when she joined the Council she was joined four years ago by another member of her sex in Dr. Janet Aitken, who is a direct representative.

It is sometimes wondered why, since Southern Ireland ceased to be part of the United Kingdom, it should still send members to the G.M.C. The situation is certainly peculiar. When the Irish Free State came into being it was mutually agreed by legislation in that country and in Great Britain that the G.M.C's constitution and powers should continue to be regulated by the British Medical Acts already in force, and it followed that the Irish Branch Council should continue as before. The direct representative is still elected by the registered practitioners in any part of Ireland; but the Crown member for Ireland is now, of course, appointed from Northern

Ireland. Doctors whose names appear in the British Medical Register are thereby entitled to practise in Eire. The Irish Free State Medical Act, 1925, set up a Medical Registration Council which maintains a Medical Register for Eire, and exercises in regard to it a jurisdiction distinct from that of the G.M.C. This close relationship, incidentally, makes it easy for newly-qualified practitioners from Irish Medical Schools to practise in this country; Eire has for long been an exporting country in this respect. It so happens that since its establishment the Irish Medical Council's President has always been a member of the G.M.C. This has been a great advantage particularly in connection with the post-war Medical Acts, and especially as regards pre-registration requirements.

### THE "MEDICAL REGISTER"

The Council's responsibility in maintaining a Medical Register is made apparent in the two volumes which are published annually and which show the state of the *Register* on 1st January. The actual *Register* itself is in the Council's office and is changing day by day; work on it occupies the whole-time attention of several members of staff. In 1959, 3,267 new names were added; 1,207 were removed—mostly owing to death; and 137 were restored after previous removal for various reasons. In addition 2,403 practitioners were placed on the provisional *Register*.

The figure of 137 names restored to the *Register* may cause some surprise. These were not all practitioners whose names had previously been erased for disciplinary reasons—in fact only three were in that category. Most of them were names which had been removed under the provisions of the Medical Acts, not on account of the death of the practitioners nor—much more unusually—at the request of doctors who had ceased to practise, but because the doctors in question could not be traced. Members of this Society whose names have been on the *Register* for five or more years will know of the provision in the Medical Acts to enable the Registrar to keep it correct. It is his duty to write periodically to each registered practitioner at his address as given in the *Register* to enquire whether he has ceased to practise or has changed his address; if no reply is received within six months the name is erased. Each year a section of the *Register* is revised in this way; and you may be surprised to hear that there are usually some hundreds of doctors who fail to reply—usually because they have changed their addresses without notifying the Registrar and without ensuring the correct forwarding of letters from their last registered addresses.

In 1955, enquiries were sent to 11,686 practitioners whose names began with the letters I to N; a quarter of these (2,900) did not reply; but after thorough investigation this number was reduced to 415 whose names were removed from the *Register*.

Bearing in mind the vital importance to a doctor of being on the *Register* it is astonishing that so many neglect to keep the Council informed when they change their addresses. When no reply is received to the Registrar's letter of enquiry he and his staff go to great trouble to try and trace the practitioner. Every possible source of information is tapped and several letters may be written; but if there is no result, off comes the name six months after the enquiry commenced.

I might instance the case of a highly esteemed practitioner in the Bristol area who a few years ago wanted to refer to his entry in the *Medical Directory*. He was disturbed at not finding it there and wrote somewhat sternly to Messrs. Churchill, the publishers. To his surprise they replied that they included in the *Directory* only the names of doctors which appeared in the *Medical Register*, and that his name did not appear there. A question to the G.M.C. revealed that his name had been erased six months after a routine enquiry from the Registrar as no reply had been received from him in spite of repeated enquiries. This man's name had been off the *Register* for, I think, about two years during which he was therefore automatically debarred from the privileges of a registered practitioner. As, however, he had been practising all this time it was fortunate that he had not come up against any legal investigation of his practice, which would certainly have made things awkward for him.

A name erased for the reason I have described can of course be restored to the *Register*; but a practitioner who finds himself in this predicament must produce, in addition to his application and a fee of £1, a statutory declaration that he is the person he makes himself out to be, together with a certificate of identity and good character from another person.

A doctor who wishes to have his name removed from the *Register* must submit a statutory declaration stating the grounds on which his application is made, and that he is not aware of any proceedings, or of any reason for the institution of any proceedings, which might result in rendering him liable to have his name erased from the *Register* by direction of the Disciplinary Committee. There is a marked contrast in this procedure with the *Dentists' Register*. A doctor's original registration fee normally covers him for life, whereas a dentist has to pay an annual retention fee; non-payment of this fee is sure to result in the removal of a dentist's name.

When one remembers also the alterations due to changes of name, registration of additional qualifications, and so on, it will be seen that the section of the Council's office which looks after the *Register* has plenty of work to do.

When first published—in 1859—the *Medical Register* contained the names of doctors possessing one or more of a number of medical qualifications specified in the 1858 Medical Act, who were engaged in medical practice in the United Kingdom at the time, and who had applied for registration. It has always been for the Council to determine which qualifications were worthy of registration. The system of visitation and inspection of examinations by the Council has been in existence in one form or another since it came into being, and has, since the 1950 Act, been supplemented by visitation of medical schools. Broadly speaking, it is on the basis of the information derived from visits and inspections that the Council decides whether a particular qualification is of a sufficient standard to justify the registration of its holders.

### OVERSEAS QUALIFICATIONS

At the beginning of its history the Council had to make decisions regarding medical qualifications awarded overseas. Inspections of qualifying examinations abroad were not at first feasible. It is of special interest to Bristolians that one of the first doctors with foreign qualifications to be registered—and that in the first edition of the *Register*—was also the first woman to have her name placed on it. Elizabeth Blackwell was born in Bristol in 1821. Her father was a sugar refiner, a man of notable moral courage who, amongst other things, believed in equality of education and opportunity for boys and girls alike. Following the accidental burning down of his refinery in 1832 he migrated with his family to the United States where, however, he died six years later, leaving his eight children penniless. Elizabeth seems to have been the strongest character in the family. After some years spent in helping to educate them she sought admission to a medical school, being determined to qualify as a doctor. After many rebuffs elsewhere she was eventually admitted by Geneva College, in the State of New York, in 1847, and after a course of only 14 months graduated in January 1849. This enterprising young woman, after studying for a while on the continent and in England, returned to New York for a few years where she practised with great success. She paid another visit to England in 1858–9 and on the strength of her American qualification and the fact that she was then practising in this country she was able to have her name entered in the *Medical Register*. After another 10 years in the United States she spent the rest of her life in England.

Elizabeth Blackwell has a strong claim for recognition as a pioneer in the medical field. She blazed the trail for a succession of women practitioners—at first a very thin stream but one which now forms a very appreciable fraction of the profession. In 1873 and 1875 the G.M.C. were faced with the question of the medical education of women in the United Kingdom; they told the Privy Council that they were "of opinion that the study and practice of Medicine and Surgery, instead of affording a field of exertion well fitted for women, do, on the contrary, present special difficulties which could not be safely disregarded"; but the Council were not prepared to say that women ought to be excluded from the profession. They considered, however, that "in the interests of public order, the education and examination of female students of Medicine should be conducted entirely apart from those of male students". One wonders what our Elizabeth thought about that; the decision of Geneva College to admit her was based largely on a unanimous resolution of the men students there "to pledge themselves that no conduct of theirs should cause her to regret her attendance at that institution".

The word "qualified", nowadays loosely used to imply having passed the final examination for a medical degree or diploma, is used in the 1858 Act quite specifically as "qualified to be registered" under the Act; i.e. from the legal standpoint registration is the important procedure. The only persons who could be registered in 1858 were the holders of British and Irish diplomas or doctors or bachelors of Medicine or Masters in Surgery of any University of the United Kingdom; holders of the Doctorate of Medicine awarded by the Archbishop of Canterbury before the passing of the Act; and "Doctors of Medicine of any foreign or colonial University or College, practising as physicians in the United Kingdom before the 1st day of October 1858, who must produce certificates to the satisfaction of the Council of having taken their degrees of Doctor of Medicine after regular examination, and who must satisfy the Council that there is sufficient reason for admitting them to be registered". It will be seen that a doctor with a foreign degree or diploma, not practising here before October 1858 had no right to be registered.

A big change occurred with the passing of the Medical Act, 1886. Part II of that Act authorised the registration of "colonial" practitioners and "foreign" practitioners holding recognized medical diplomas, able to satisfy the Council that they were of good character, and entitled by law to practise Medicine, Surgery and Midwifery in the country in which they obtained their medical qualification. The definition of "a recognized medical diploma" was "such a diploma as may be recognized for the time being by the General Council as furnishing a sufficient guarantee of the possession of the requisite knowledge and skill for the efficient practice of medicine, surgery and midwifery"; this definition holds good in exactly the same terms today for all primary qualifications which are registrable. But there was an over-riding section to the effect that the registration of overseas practitioners should not apply to any British possession or foreign country until the Sovereign by Order in Council should declare that in his opinion such possession or country "affords to registered medical practitioners of the United Kingdom such privileges of practising as may seem just". The only amendment of these provisions in the 1950 and 1956 Medical Acts has been the substitution of the word "Commonwealth" for colonial".

It will be seen, therefore, that registration of practitioners with overseas medical qualifications depends fundamentally on the principle of reciprocity between the United Kingdom and other countries and on the recognition by the G.M.C. of the adequacy of the overseas degrees or diplomas held by applicants for registration. This latter condition involves the responsibility of the Council to decide whether a particular qualification is a sufficient guarantee that its holder possesses sufficient knowledge and skill for the practice of his profession. This is naturally a great responsibility, particularly when it is difficult for the Council to obtain its own first-hand evidence of the standard of education and examinations in overseas universities and colleges. It forms an important part of the work of its Executive Committee, which at its meetings nowadays commonly has to devote a substantial amount of time to questions of recognition. Opportunities have been taken from time to time for members of the Council, or deputies appointed by it, to visit overseas medical schools and their examinations. During the past few years a good deal of time has been devoted to applications from medical institutions in India and Pakistan.

Reciprocity at present exists between this country and—in Australia—New South Wales, Queensland, South Australia and, very recently, Western Australia; in Canada—Alberta, Manitoba, Newfoundland, Nova Scotia, Prince Edward Island, and Saskatchewan; Ceylon, Hong Kong, India, Malta, New Zealand, Pakistan, Singapore and Malaya, South Africa, Uganda and one foreign country, Burma. Not all of the degrees and diplomas in some of these countries are recognized as adequate qualifications for registration. Unfortunately the United Kingdom has no reciprocity with certain Commonwealth countries.

The recent distinction between provisional registration and full registration necessitated by the 1950 Act is, of course, applicable also to holders of overseas medical degrees and diplomas. The G.M.C. now has to decide whether these practitioners have had experience "not less extensive" than the year's experience in hospital posts required of British graduates, before they can be fully registered. This work is a heavy burden on the time of the four members of the Council's Commonwealth and Foreign Registrations Committee who weekly receive numbers of applications for decision.

During the last War temporary registration was available; and since 1947 the G.M.C. has been empowered to grant similar registration to any overseas practitioner who has passed a qualifying examination recognized by the Council as sufficient and who intends to be temporarily employed in a professional capacity in a United Kingdom hospital. Apart from practitioners who are eligible for provisional or full registration, about 500 doctors avail themselves annually of this form of registration, which is some indication of the extent to which these islands are a centre of medical postgraduate education for citizens of foreign countries as well as Commonwealth practitioners.

The Commonwealth section of the *Medical Register* is quite an appreciable fraction of the whole; at the beginning of 1960 it contained 12,183 names—and the Foreign section 1711—out of a total of 92,889.

### UNQUALIFIED PRACTITIONERS

As I have already mentioned, the fundamental object of the Medical Acts has always been to assist and protect the public. This aim would, of course, be nullified if the elaborate procedure before a doctor's name is placed on the *Register* were undermined by people, however well-meaning, making out that they were registered medical practitioners when in fact they were not. The General Medical Council itself has no control over those who are not on its *Register*, but it usually gets to hear when there has been a successful prosecution of a person for falsely pretending to be a physician, surgeon or general practitioner, or falsely implying that his name is on the *Register*—for which the maximum penalty now is £500. The latest of such successful prosecutions which is recorded in the Council's minutes was that of an unregistered man who called himself a "Homoeopathic Medical Practitioner and a certified Physico-medical Botanic Physician". Unregistered persons often describe themselves in very weird ways.

There was an unusual case in Bristol not many years ago which came to my notice locally before the G.M.C. heard of it. A medical auxiliary with a burning ambition to be a doctor was sent down by the University for disciplinary reasons before he had taken the first M.B. examination. He then spent a good deal of time studying medical text-books from which he appears to have amassed considerable theoretical knowledge. On seeing an advertisement for a locum in general practice owing to the practitioner's serious illness he was able to secure the post; although his name was not of course on the Medical Register, a fact which local authorities pointed out, he was able to produce a typewritten letter on G.M.C. stationery and signed by the Council's Chief Clerk, which stated that his name had been omitted from the *Register* in error and that he was in fact registered.

Some time previously this man had received a letter from the Council's office giving him advice regarding entry to medical schools; he had erased the text of the letter itself without interfering with the signature and had skilfully typed in his own wording. This man "practised" as an assistant for some months, taking care to avoid prescribing dangerous drugs over his own name, midwifery cases and so on, and to judge from his patients' testimony he must have developed an excellent bedside manner and he appeared to inspire complete confidence. His one really careless error was to set up in this way in his home town and not many miles from the University, some of whose staff remembered him as an unsuccessful student in the first medical year.

### THE BRITISH PHARMACOPOEIA

The third main duty imposed on the G.M.C. by the 1858 Act was the publication of the *British Pharmacopoeia.* The Royal Colleges of Physicians in London, Edinburgh and Dublin had previously published independent pharmacopoeias, and it was clearly desirable to have one standard list of drugs for these Islands. The Council set about this formidable task, but the first *British Pharmacopoeia*, which appeared six years after the foundation of the Council, was found to contain so many imperfections that a second edition had to be published three years later. A third *Pharmacopoeia* was published in 1885, a fourth in 1898, and a fifth in 1914.

At first it was a committee of members of the Council themselves who prepared the *Pharmacopoeia.* As time went on, the lack of ability of individual members for this specialized work resulted in the extensive use of outside experts. Following the recommendations in a report issued by the Committee on Civil Research in 1928, a British Pharmacopoeia Commission was established to draft amendments and additions to the *Pharmacopoeia*; it now consists of eleven authorities in medicine, therapeutics, pharmacology, pharmacy and pharmaceutical chemistry, appointed by the Council on the recommendation of a representative selection committee. The Commission holds regular meetings, and work is continually proceeding by sub-committees—now numbering twenty-one—which are composed of experts in particular aspects of the *Pharmacopoeia.* It has its own laboratory and permanent secretarial officers.

The Council has a standing Pharmacopoeia Committee to which the Pharmacopoeia Commission reports and which, on the Council's behalf, gives general directions to the Commission. It is the duty of the Council to publish the *Pharmacopoeia*, and through its Committee it gives final approval to the draft submitted by the Commission. The first *Pharmacopoeia* published under these conditions appeared in 1932. The Second World War delayed the publication of the next *Pharmacopoeia* until 1948; and such was the progress of pharmaceutical and therapeutic knowledge that the Council then decided that the intervals between editions should be reduced to five years, with an Addendum appearing about half-way between the main publications; one such Addendum was published on 3rd October, 1960.

On the publication of a new edition the Commission which prepared it goes out of office, and a new Commission, appointed in the same careful way and usually including several members of the previous body, commences work for the next edition.

The greater part of the *British Pharmacopoeia* consists of monographs dealing with individual drugs and the official preparations containing them. The 1858 Act defined it as "a book containing a list of medicines and compounds, and the manner of preparing them, together with the true weights and measures by which they are to be prepared and mixed, and containing such other matter and things relating thereto as the General Council shall think fit". The 1950 Act spoke rather more concisely: "containing such descriptions of, and standards for, and such notes and other matter relating to, medicines, preparations, materials and articles used in the practice of medicine, surgery or midwifery as the Council may direct". The *Pharmacopoeia* also contains an important section of methods for carrying out biological assays on drugs which cannot appropriately be standardized in any other way. It is not, of course, a text-book of therapeutics, and many doctors no doubt have never seen it; but it is the constant companion of the pharmacist and it is essential for the pharmaceutical manufacturer; for the student of pharmacy it is practically a bedside book. To the medical practitioner it is a guarantee that when he prescribes a drug or preparation included in it he can rely on his patient obtaining an article of the quality and character therein defined.

The 1953 *Pharmacopoeia* was the first in which the main titles of drugs and their preparations were given in English instead of Latin. Weights and measures were originally given only in the Imperial system; but before the close of the last century the metric system had begun to creep in, and in the 1963 *Pharmacopoeia* all references to the Imperial system will probably be removed.

Since the War the great development of the pharmaceutical industry has resulted in the production of very many new drugs which are introduced under the makers' proprietary names, with the result that a given drug may be marketed under several different names. In order to reduce confusion the British Pharmacopoeia Commission periodically issues lists of new non-proprietary names which can be used by any manufacturer and which, if the drugs in question are subsequently described in the *Pharmacopoeia*, will become the official titles. These lists contain in a second column, opposite the new names, the various proprietary names so that reference is relatively easy. The lists of new names are circulated for approval to the Council's Pharmacopoeia Committee whose wonder at the number and complexity of the new names can only be matched by their admiration of the ingenuity with which the names have been derived from the chemical nature of the new drugs. Amongst the criteria which the Commission adopt in inventing these new words are: the name should be as short as is convenient, distinctive in sound and spelling, not liable to be confused with existing names nor difficult to pronounce or remember, and free from anatomical, pathological, pharmacological, physiological or therapeutic suggestion; an attempt is always made to form the new name by a combination of letters or syllables from the scientific chemical name. A few of these names are of course well-known to practitioners, but one almost hopes that a lot of the others will never find their way into the *Pharmacopoeia*!

### PRESIDENTIAL WARNINGS

There are certain ways in which doctors owing to the nature of their profession are peculiarly liable to fall foul of the law. One of these is in connection with legislation and regulations on Dangerous Drugs. Every year the Council is notified by the Home Office of practitioners whose authority to prescribe these drugs has been withdrawn and some of these cases of course are considered by the Disciplinary Committee. Among the most serious are those of doctors who, on prescriptions ostensibly for their patients, obtain large quantities of drugs of addiction for their own use. On at least two occasions within my experience the Home Office has felt obliged to ask the Council to warn the profession against carelessness in ensuring the safe custody of drugs in their possession—on one occasion in particular against leaving dangerous drugs in unattended and unlocked cars.

Warnings concerning these and other matters are introduced into the addresses which successive Presidents have made to the Council at the beginning of each session and you may perhaps remember a pronouncement by the present President in 1953 which was challenged in certain quarters as a slur on the profession. It might be of interest if I deal briefly with the history of the matter.

The last War added heavily to the commitments of doctors (who were in depleted numbers) by the numerous certificates which they were required to sign, in appropriate circumstances, for their patients. Lax certification has always been a subject specifically mentioned in what used to be called the "Warning Notice" issued by the Council; but during the War certain flagrant examples of not lax, but deliberately fraudulent certification came to light. In any war, presumably, there will always be a small proportion of men who for reasons of their own, other than their consciences, will try their best to evade service in the Armed Forces of the Crown, and medical grounds form an obvious loophole. Some of these people by giving false histories of epilepsy were able to persuade doctors on those grounds to certify them as unfit for service. In most of these cases the doctors in question benefitted greatly by the large sums of money which changed hands when they handed over their certificates to these bogus epileptics, and on at least one occasion evidence was obtained to the effect that the practitioner had actually instructed his patients on how to describe epileptic fits. Needless to say, the Council had no hesitation in erasing from the *Register* the names of those who had been found guilty of such unprofessional conduct.

Not long after the need for special wartime certificates had passed, the National Health Service came into being, and with it more certification of different kinds. Obtaining sickness benefit without valid cause is an objective of some unscrupulous people who either personally or by relatives can persuade their doctors to certify them as being unfit for work without adequate examination or without even having seen the patient. Obtaining money from an Executive Council by wrongly submitting forms which purport to relate to the treatment of temporary residents who in fact had not been treated is another cause of disciplinary action. Such cases became so frequent that the President regarded it as his duty to refer to the matter in one of his addresses in the following terms: "I desire, therefore, to take this opportunity—and I do this with the consent and on the advice of the Disciplinary Committee—of bringing to the notice of the whole profession the gravity of offences of this nature".

This was unfortunately, and quite unreasonably, construed by a few doctors as being a grave slur on the whole profession. In his next address the President denied such intention. "In my address last November I expressed the concern of the Disciplinary Committee at the emergence of certain professional misdemeanours in connection with the National Health Service which had kept recurring during the past few years, and on their advice issued a warning. I have since learned that this warning has been interpreted in some quarters as indicating that these misdemeanours are widespread and as a reflection on the high general ethical standard of the profession in Great Britain. This was neither intended nor implied. The Minister of Health, when asked in the House of Commons on 21st January as to what steps he had taken since my statement was made, said 'I have taken no steps since the statement made by the President of the General Medical Council, but it is fair to say that perhaps the right interpretation has not been put on what was said. As I understand, it was a warning that the General Medical Council rightly take a grave view of laxity in these matters, and was not an allegation that fraudulent practices or conduct were at all widespread.' "

### MISCELLANEOUS FUNCTIONS

The Council is often invited to give evidence to Commissions, Committees, and Government Departments on matters within its competence. It is one of the bodies frequently approached by the Board of Trade for observations on applications under the Companies Act, or when medical or related bodies are applying for Charters of Incorporation. The Institute of Cancer Research, the British Student Tuberculosis Foundation, the Association of Clinical Pathologists, the Institute of Basic Medical Sciences, the British Orthoptic Society, the College of Speech Therapists, the Birmingham and Merseyside Blood Transfusion Services, the Electroencephalographic Society, and the National Association for the Paralysed are recent examples of bodies wishing to dispense with the word "Limited" in their titles. Occasionally the Council's advice is sought in connection with an application from a number of practitioners who desire to band themselves together in a Company with a name such as "Dr. X and Partners". There usually seems to be no objection to the omission of the word "Limited" after the name of such a Company.

In 1951 the World Medical Association recommended that the Declaration of Geneva, replacing the Hippocratic Oath, should be made by all medical students when qualifying. This recommendation was referred by one of the British Licensing Bodies to the G.M.C. Suitable though the principles of this Declaration would be to place before students during their training, very few Licensing Bodies in the British Isles in fact require their students to subscribe to it or to the Hippocratic Oath, and it is therefore not surprising to find the Council replying that "It falls outside the scope of their statutory responsibilities to express an opinion whether Licensing Bodies should require, or invite, their newly qualified students to make declarations or affirmations before qualifying diplomas are conferred upon them".

When to the many activities I have described we add the disciplinary business, the preparation of which may be very heavy, and the educational side of the Council's functions it will be realized that each day brings a lot of new work into the Office. Many items require the personal attention of the President, and it is usual for the Register, or Assistant Registrar, to prepare them for him with a carefully drafted minute giving relevant facts and precedents which to a considerable extent eases his burden. This can only be done with a first-rate staff, the Registrar himself being indispensable. During my time on the Council I have known two Registrars; the more one sees of the work and accomplishments of the holders of that office the more one is filled with admiration for their consummate wisdom and ability. The Registrar is in almost continuous touch with the President; yet he is always in the background, so that no one who has had no personal experience of the Council's work can have any conception of the extent to which the smooth prosecution of its business depends on his quite invaluable services.

While not wishing to make this an educational address I hope that, in spite of its discursive nature, I may have succeeded in imparting some interesting information about the Statutory Body which, though established for the benefit of the public in medical matters, is yet of great importance to the medical profession itself.

